# The Ohio State Dental Society

**Published:** 1894-01

**Authors:** 


### The Ohio State Dental Society.

The annual meeting of the Ohio State Dental Society was held in the Neil House, Columbus, December last, 5th, 6th and 7th.

There was present a larger number of the membership than for several years, and a warm interest was manifested in its work and prosperity. Quite a number of papers were read and discussed, which will appear in the next number of the REGISTER. The papers were all good. We would direct attention especially to the papers of Drs. J. R. Callahan and G. Molyneaux. The former on "The Use of Sulphuric Acid in Opening and Cleansing Pulp-Chambers and Root-Canals." The process was certainly new to many, and in the discussion which followed, and the demonstrations presented, great interest was manifested. It is a method of procedure, for the purpose specified, that will certainly call forth special attention from the profession, and doubtless will add very greatly to the facility in this line of work. Opening and cleansing and putting in proper condition root-canals of pulpless teeth has to the great proportion of the profession, at least, been an operation attended by very great difficulty, and many a time with uncertain success. According to the experience of Drs. Callahan and Heise, extending over three or four years, it is pretty conclusively shown that more can be accomplished by this method of procedure than by any that had preceded it.

The paper of Dr. Molyneaux on "Arranging and Articulating Artificial Teeth, especially full Dentures, with the Articulator devised by Dr. Bonwill" showed marked progress in this direction. By nearly all methods of articulating artificial teeth heretofore employed, or at least, prior to the introduction of Dr. Bonwill's method and appliance, there was a lack of scientific precision and accuracy that seemed very desirable. The difficulties that attended the arrangement and articulation of artificial teeth seem to be, by the intelligent use of this method, completely overcome.

It is a matter of regret, however, that the discussions upon these papers, as well as upon the others, were not fully reported.

This we regarded at the time, and still regard as a great oversight on the part of the Executive Committee of the Society. We are promised faithfully, however, that this lapse will not again occur. This society is old enough, has done good work enough to have its doings fully recorded, and the profession thereby enabled to derive the benefit from these proceedings. The interest manifested in this meeting is certainly indicative of a more general interest in the work of the society. A large number of new members were added, and one very encouraging feature was that the younger members of the society were inclined and actually did take hold of the work better than ever before. If the profession generally could realize how much strength and encouragement is imparted by attendance at these meetings, the numbers would be vastly increased and more encouragement and stimulus given to all.

We think the hope is fully warranted that the meeting of 1894 will be the largest and the best that has ever been held.

